# The Effect of Thermal-Treating on Drug Release from Sustained Release Alginate-Eudragit RS Matrices

**DOI:** 10.34172/apb.2021.027

**Published:** 2020-04-19

**Authors:** Ehsan Kaffash, Mohammadreza Abbaspour, Hadi Afrasiabi Garekani, Zohreh Jahanian, Farinaz Saremnejad, Abbas Akhgari

**Affiliations:** ^1^Department of Pharmaceutics, School of Pharmacy, Mashhad University of Medical Sciences, Mashhad, Iran.; ^2^Student Research Committee, Mashhad University of Medical Sciences, Mashhad, Iran.; ^3^Targeted Drug Delivery Research Center, Pharmaceutical Technology Institute, Mashhad University of Medical Sciences, Mashhad, Iran.; ^4^Department of Food Science and Technology, Ferdowsi University of Mashhad, Mashhad, Iran.

**Keywords:** Alginate, Calcium, Eudragit^®^ RS, 5-aminosalicylic acid, Pellet, Curing

## Abstract

***Purpose:*** The main objective of the present study was to develop the colonic delivery system for 5-aminosalicylic acid (5-ASA) as an anti-inflammatory drug.

***Methods:*** Matrix pellets containing various proportions of alginate, calcium and Eudragit^®^ RS were prepared by extrusion-spheronization technique. Thermal treatment was used to investigate the effect of the curing process on the surface morphology, mechanical and physicochemical properties and *in vitro* drug release profile of pellets. Based on the obtained results optimal formulations were selected to coating by the Eudragit^®^ RS and subjected to a subsequent continuous dissolution test.

***Results:*** Image analysis and also scanning electron microscopy results proved acceptable morphology of the pellets. The fourier transform infrared spectroscopy and differential scanning calorimetry studies ruled out any interactions between the formulation’s components. Curing process did not alter the mechanical properties of pellets. The release rate of the drug from matrices was prolonged due to the decreased porosity of cured pellets. Furthermore, selected cured pellets which coated with Eudragit^®^ RS, prevented undesired premature drug release.

***Conclusion:*** Formulation containing 17.5% calcium, 17.5% alginate, and a coating level of 10% demonstrated enhanced drug release so that provided resistance to acidic conditions, allowing complete drug release in alkaline pH, mimicking colonic environment. The slow and consistent drug release from this formulation could be used for treatment of a broader range of Inflammatory bowel disease (IBD) patients especially in whom colonic pH levels have been measured at lower than pH 7.0.

## Introduction

Inflammatory bowel disease (IBD), which caused the inflammation of the gastrointestinal tract (GIT), consists of two groups, including Crohn’s disease and ulcerative colitis. GIT is the main site of pathology in IBD patients, so the oral route has the potential to deliver the drug to the site of action.^1^ Corticosteroids are potent anti-inflammatory therapies, but their long-term use is often caused severe systemic side effects such as hypertension, hyperglycemia, and immunosuppression.^2^ Therefore, 5-aminosalicylic acid (5-ASA) is still considered as first-line therapy for mild to moderate types of IBD. However, 5-ASA is slightly soluble in water, at 25°C (USP-NF), and its oral administration is resulting in low bioavailability due to rapid and extensive absorption on the upper GIT.^3,4^

Therefore, optimizing the drug delivery systems is necessary for avoiding the systemic side effects as well as improving the local bioavailability of 5-ASA at target inflamed tissues. until now, a wide range of oral colon targeted drug delivery systems have been investigated. pH, time, microflora, and pressure-dependent systems are the primary drug delivery approaches in IBD therapy.^5^ These modified-release drug systems achieved by applying natural and synthetic polymers as a matrix or a coating layer of a single unit or multi-particulate delivery system. Matrix drug delivery systems include hydrophobic and hydrophilic types. Hydrophilic matrices are widely used for sustained drug release in GIT. The swelling and eroding behavior of these matrices in an aqueous medium provides the potential to acquire a suitable dissolution profile.^6^

Alginate is a natural anionic polysaccharide that contains two uronic acids, α-L-guluronic and β-D-mannuronic acids.^7^ Due to compelling characteristics such as non-toxicity, biocompatibility, biodegradability, and the ability to form a gel with multivalent cations, alginate have several applications in drug delivery and controlled release systems.^8-10^ It has also been used to produce hydrophilic matrices for oral controlled release dosage forms.^11^ Alginate solutions form a hydrogel matrix via the exchange of sodium ions from guluronic acid with divalent ions, such as magnesium, calcium, and etc.^12^ However, the complete erosion which leads to faster drug release from the matrix is still a significant challenge.^13^

**Figure 1:**
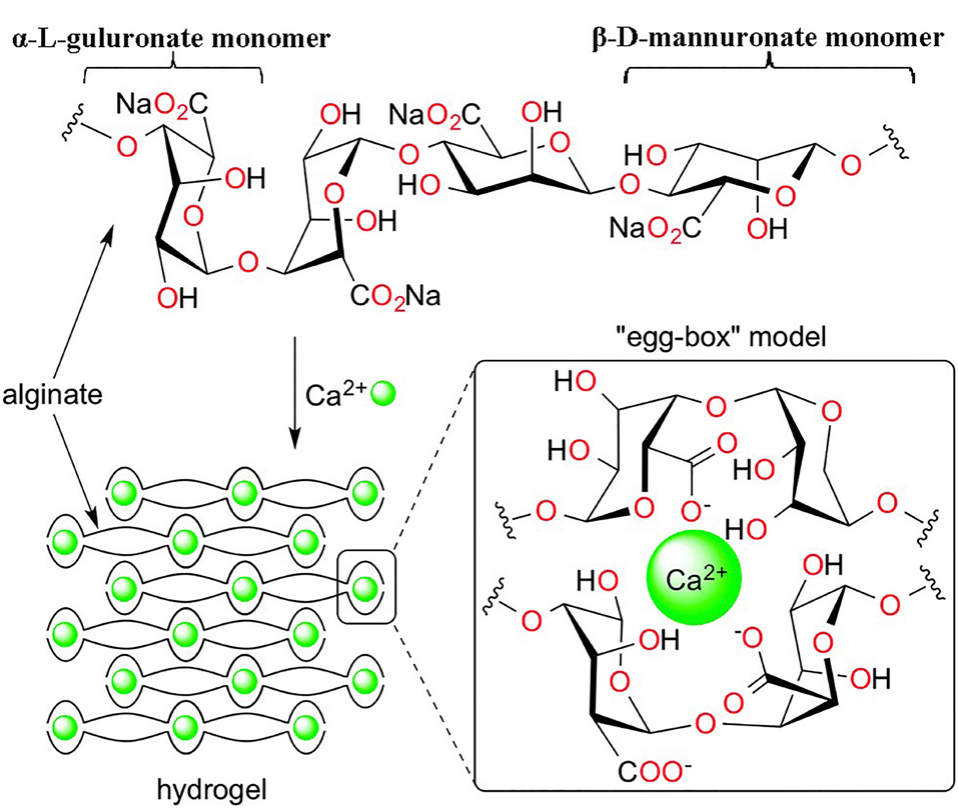
Structure of alginate and hydrogel formation by incorporation of Ca^2+^ (the “egg-box” model adapted from ^21^).

Adding calcium salts to the matrix could lead to the formation of a calcium-alginate complex, which provided an excellent carrier in the delivery system of the active agent. This process happens by cross-linking of the calcium ions with uronic acids of alginate.^14^ Each calcium ion takes part in co-ordination link with an oxygen atom, resulting in a three-dimensional network of calcium-alginate popularly known as the “egg-box model” (Figure 1).^15^ However, the calcium-alginate complex might show some restrictions, such as non-controlled swelling properties and drug release profiles.^12^ In this way, the combination of this biopolymer with methacrylic acid and methacrylate polymers (Eudragit) increased the efficiency of the drug delivery system in terms of reaching the majority of the drug to the colon.^16,17^ Eudragit^®^ RS has been efficiently representing bifunctional release characteristics, i.e., time-dependent and site-specific (such as the colonic site).^18^ So, using a water-insoluble polymer such as Eudragit^®^ RS in combination with an anionic polymer could reduce the permeability of the enteric matrix pellets during the passage of acidic medium. Microcrystalline cellulose (Avicel) is known as a filler and binder agent in multiparticulate formulations, which helps to achieve desire sphericity and shapes.^17^

Also, it has been shown that the thermal treatment or curing process reduces the diffusion rate of ions (e.g., Ca^2+^), which controlled the gel-forming of Alginate.^19^ The drug release rate may be significantly altered by curing the polymeric matrices above the glass transition temperature.^20^ Also, curing can prolong the release rate of the drug from matrices due to the decreased porosity.^20^

In the current study, the aim was to design pH and time-dependent matrix systems. It is hypothesized that curing procedure could control the swelling and, consequently, decrease initial burst release of drug from the matrix and provide an appropriate method to attain a sustained release of 5-ASA from the alginate-based matrices. The extrusion-spheronization method was used to prepare matrix pellets. Also, formulations with the different weight percentages of calcium, Alginate, and Eudragit were prepared. Differential scanning calorimetry, Fourier transform infrared spectroscopy, mechanical tests, scanning electron microscopy, image, and sieve analysis were carried out to investigate the physicochemical properties of prepared pellets. Furthermore, dissolution studies were performed in different pH situations similar to simulated gastrointestinal media due to evaluate the drug release behavior of optimized formulations.

## Materials and Methods

### Materials

5-ASA was prepared from Iran Najo co. (Iran). Microcrystalline cellulose (Avicel^®^ PH101) was provided by Samisaz (Mashhad, Iran). Calcium acetate and potassium phosphate monobasic were obtained from Sigma (China). Sodium alginate (Mw 80–120 kDa with mannuronate/guluronate ratio of 1.56) was purchased from Sigma-Aldrich (USA). Eudragit^®^ RS PO (Mw ∼150 kDa) was provided from Evonic^®^ Degussa (India Pvt. Ltd., Mumbai, India). All other chemicals, reagents, and solvents were of the highest commercially available analytical grade.

### Methods

#### Preparation of pellets

Dry formulation components (Table 1) were mixed for 8 min. Then, due to getting moist mass with proper consistency, a sufficient amount of water as a granulating agent was added to the mixture. Plastic wet mixture through the axial extruder (Malvern, UK) was passed with a die of 1 mm thick with perforations of 1 mm in diameter and operated at speed 100 rpm. The extrudates were rounded by a spheronizer (Dorsa HC 732, Iran) with a cross-hatched plate at 1000 rpm for 2 minutes. Half of the obtained pellets were cured at 50°C in an oven (Parseh, Iran) for 5 hours, and the remained part was dried at room temperature.^22^

**Table 1:** Percentage ofpellets formulation components

**Formulation**	**Eudragit** ^®^ ** RS**	**Calcium acetate**	**Alginate**	**5-ASA**	**Avicel**
F1	0.0	30.0	30.0	20.0	20.0
F2	7.5	7.5	45.0	20.0	20.0
F3	7.5	45.0	7.5	20.0	20.0
F4	25.0	0.0	35.0	20.0	20.0
F5	25.0	17.5	17.5	20.0	20.0
F6	25.0	35.0	0.0	20.0	20.0
F7	42.7	2.5	14.8	20.0	20.0
F8	42.7	14. 8	2.5	20.0	20.0
F9	50.0	5.0	5.0	20.0	20.0

#### Coating of pellets

30 % (w/v) solutions of polymer Eudragit^®^ RS PO were prepared in isopropyl alcohol: water (9:1) mixture. Triethyl citrate was added to the solution as a plasticizer (10% (w/w) related to dry polymer). Talc also was added as a glidant (5% (w/w) related to dry polymer. The resulted suspension was coated onto 100 g of F1, F5, and F8 formulations using fluidized bed coater (Werner Glatt, Germany). The inlet air temperature was set at 40^°^C, and the outlet temperature was in the range of 25–35°C. Samples of coated pellets were removed from the apparatus when the coating loads reached 5 and 10 % (w/w) weight gain. At each stage, the pellets were fluidized for extra 5 min, and samples were kept in an oven for 2 hours at 40°C.^23^

#### Sieve analysis

Pellets were sieved using standard 18 and 35 mesh sieves. The weight of the remaining pellets was then determined on each sieve. The remained pellets on the mesh 35 sieve (size range 500-1000 μm) were considered as appropriate pellets. This was performed for all formulation of pellets. The weights and yield of pellets were reported.

#### Image analysis

For this test, images were obtained using a Nikon^®^ camera coupled to a Motic SMZ 168^®^ optic microscope (Motic Incorporation Ltd) with a 10X/23 eyepiece. Measurements were performed with the aid of ImageJ^®^ (version 50) software, using images of 8× magnification with pellets ranging from 500 to 1000 μm. Aspect ratio was calculated by dividing the value of the largest diameter of the pellet by the perpendicular diameter. Also, to obtained sphericity the area (A) and perimeter () were measured, then it was calculated as followed^24,25^:

(1) $$Sphericity=\frac{4\pi A}{P_{m}^{2}}$$

#### Scanning electron microscopy (SEM)

Surface and morphology characteristics of pellets were studied by SEM (TESCAN FE-SEM MIRA3, England). SEM was performed on pellets after and before dissolution tests. The pellets were arranged in a thin layer of silver for 10 minutes under argon gas, then the samples were examined by an electron microscope, and the images were recorded.

#### Mechanical tests

For the mechanical testing, 20 pellets in size range of 500–1000 µm were tested using Testing Machine (Hounsfield, England). Force-displacement graphs were obtained by a computer system attached to the apparatus (QMAT, Hounsfield, England). The crushing strengths (CS) of pellets were obtained directly from the plot and elastic modulus of pellets were determined from the slopes of the plots as described previously.^26,27^ To compare the mechanical properties of the uncured and cured samples, the unpaired *t* test was used by Prism GraphPad software version 6.01 at a significance level of α = 0.05.

#### Differential scanning calorimetry (DSC)

Thermal characteristics of formulations’ components were determined using an ATA449-C instrument (NETZSCH, Bavaria, Germany). The samples were heated from 0 to 250°C at a heating rate of 10°C/min.

#### Fourier transform infrared spectroscopy (FTIR)

The spectrometer was recorded at 400 to 4000 cm^-1^ wavelengths. The homogeneous powder of the raw materials and formulations was prepared, and then their absorption diagram was directly recorded by using IR spectroscopy equipment (Thermo Nicolet, Madison, WI, USA).

#### Dissolution study

In vitro studies of uncoated (cured and uncured) and coated pellets were carried out by a USP apparatus I (basket method) in the 900 mL medium at 37°C at a rotation speed of 100 rpm (Shanghai Huanghai Instrument Co., China). The pellets were accurately weighed containing the equivalent of 50 mg of 5-ASA were transferred to the basket. The dissolution tests were done in pH 1.2 (HCl 0.1N) and phosphate buffer solution pH 6.8, respectively.

The absorption of the samples was recorded at a wavelength of 302 nm (for acidic medium) and 330 nm (for buffer medium) spectrophotometrically. From the absorbance readings, cumulative percentage of drug dissolved was calculated. The dissolution test was performed for 2 hours for the acidic medium (pH 1.2) and 10 hours in the buffer medium (pH 6.8).

In order to compare the different drug release diagrams, an independent method was used, calculating the mean dissolution time (MDT) allows direct comparison of the data from the dissolution test. In this way, all the dissolution data is expressed as a parameter. MDT was calculated by the following equations^28^:

(2) $$MDT=\frac{\sum {\bar{t}}_{i}.\Delta M_{i}}{\sum \Delta M_{i}}$$

(3) $${\bar{t}}_{i}=\frac{t_{i}+t_{i+1}}{2}$$

(4) $$\Delta M_{i}=M_{i+1}-M_{i}$$

Where ${\bar{t}}_{i}$ is the midpoint of the time during which the fraction $\Delta M_{i}$ of the drug released from the dosage form. A high MDT value shows that the drug delivery system has more resisted in drug release during in vitro studies.

The continuous dissolution test was carried out for further confirmation based on accepted GIT times; 2 hours for pH 1.2 and 10 hours for pH 6.8, respectively.

## Results and Discussion

### Shape characteristics of pellets

Based on Table 2 results, the size distribution analysis by sieving indicated that in the range size of 500-1000 µm, the yield of pellets was higher than 80%. Also, image analysis results showed that the sphericity of the most formulations was higher than 0.7, which indicates that the pellets are almost spherical. The aspect ratios were acceptable, i.e., ≈1.3 (±0.2 SD) for all formulations which were considered acceptable in many studies.^29,30^ The lowest yield of pellets and sphericity, as well as the highest aspect ratio associated with the F4 formulation. F4 does not have calcium but has a high amount of alginate. Alginate alone causes the pellets to exhibit elastic behavior and lose their uniformity and sphericity. Similarly, some studies reported that the use of alginate in formulation affects pellets size^31^ and by changing the plasticity, creates dumbbell-shaped pellets.^32^ Calcium ions are able to make cross-link with the sodium alginate and reduce its swelling.^33^ Therefore, the addition of calcium acetate to the formulations, which containing sodium alginate, would reduce the pellet size and makes them more spherical.

**Table 2:** The results of the morphological and mechanical characteristics of the pellets

**Formulation**	**The yield of pellets (%)**	**Sphericity**	**Aspect ratio**	**Hardness (N)**		**Elastic modulus (MPa)**	
				**Uncured**	**Cured**	**Uncured**	**Cured**
F1	79.74	0.74±0.11	1.37±0.22	4.0±0.8	2.5±0.6	0.6±0.1	0.4±0.1
F2	92.93	0.69±0.14	1.51±0.39	3.6±1.0	3.7±1.1	0.4±0.1	0.6±0.3
F3	58.17	0.76±0.09	1.33±0.16	5.6±1.7	4.0±0.8	1.0±0.2	0.7±0.1
F4	57.90	0.56±0.14	1.56±0.54	10.7±0.6	15.7±2.7	1.0±0.2	1.1±0.3
F5	94.27	0.73±0.12	1.41±0.26	5.3±1.6	6.0±1.2	0.8±0.3	0.9±0.2
F6	81.26	0.82±0.08	1.24±0.14	5.9±0.5	5.0±1.1	1.1±0.3	1.0±0.2
F7	94.23	0.65±0.09	1.54±0.22	7.9±3.4	12.1±2.8	0.8±0.1	1.2±0.3
F8	91.40	0.75±0.10	1.37±0.2	4.6±1.1	4.3±1.4	0.5±0.1	0.6±0.1
F9	85.66	0.78±0.12	1.35±0.23	3.9±0.8	4.6±0.8	0.4±0.8	0.5±0.1

**Figure 2:**
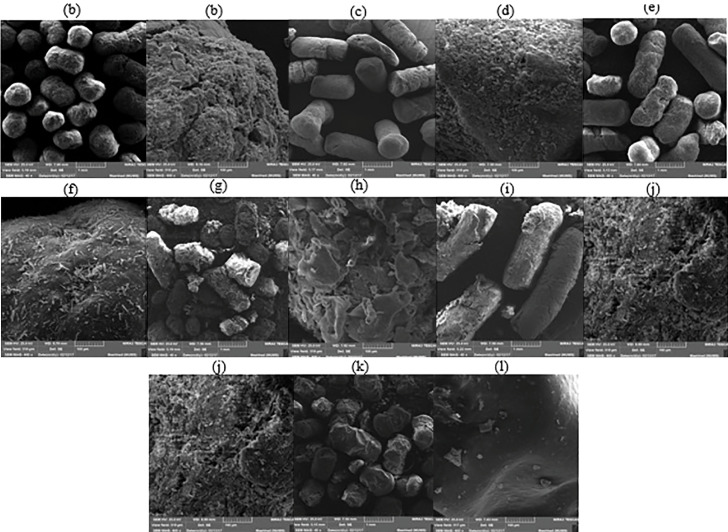
SEM images of pellets: uncured at 40× (a) and 400× (b), cured at 40× (c) and 400× (d), 10% coated at 40× (e) and 400× (f), cured after dissolution in 0.1 N HCl at 40× (g) and 400× (h), cured after dissolution in phosphate buffer 6.8 at 40× (i) and 400× (j), 10% coated after dissolution in 0.1 N HCl at 40× (k) and 400× (l), 10% coated after dissolution in phosphate buffer 6.8 at 40× (m) and 400× (n).

F5 was selected to SEM imaging (Figure 2) in order to study the surface properties and then to observe the effect of Eudragit^®^ RS coating on prepared pellets. In 40× magnification of these images, the uniformity of the shape and surface of the pellets is apparent as well as in larger magnification (400×), the crystals of the drug are visible. In the cured pellets (Figure 2c, d), the surface is smoother than the uncured pellets (Figure 2a, b) due to the polymer melting and placement of the drug particles and Avicel in the melted polymer matrix. The uniform surface of the coated pellets covered the crystals of the drug (Figure 2e). The observed particles on the surface of the pellets (Figure 2f) relate to the talc, which is uniformly positioned on the coating. Figure 2g, h shows swelling of the cured pellets after the dissolution test in 0.1 N HCl. However, after the dissolution test in the phosphate buffer 6.8 (Figure 2i, j), surface pores can correspond to the loss of polymeric network integrity which leads to drug release. In the case of the coated pellets after the dissolution test in 0.1 N HCl (Figure 2k, l), the coating remained intact which demonstrates the drug release through the coating layer occurred by the diffusion mechanism. The small channels caused by swelling of Ca-alginate resulted in drug diffusion.^34^ After the dissolution test in the phosphate buffer 6.8 (Figure 2m, n), pores appeared in the coating layer. These pores may be created due to the higher swelling of the Ca-alginate in the buffered medium.^34^

### Mechanical properties

Results showed that there was no significant difference between the mechanical properties of cured and uncured pellets. However, F4 and F7 formulations have higher hardness and elastic modulus than other formulations and also curing process led to a further increase in these parameters (Table 2). In F4, due to a high amount of alginate, a very sticky wet mass produced very hard pellets after drying. In the case of the F7 formulation with high Eudragit^®^ RS, the structure of the pellets has become more coherent, and the elastic modulus has increased due to the melting of the Eudragit^®^ RS during the curing process. Increasing the mechanical strength of matrix pellets containing a high amount of Eudragit due to curing has also been proven in other studies.^20,35^ On the other hand, Eudragit^®^ RS-free F1 formulation was showed lower hardness and elastic modulus after the curing process. It can be due to the water loss of the pellets, which increases pellets’ fragility by reducing the plasticity.

### DSC characterization 

**Figure 3:**
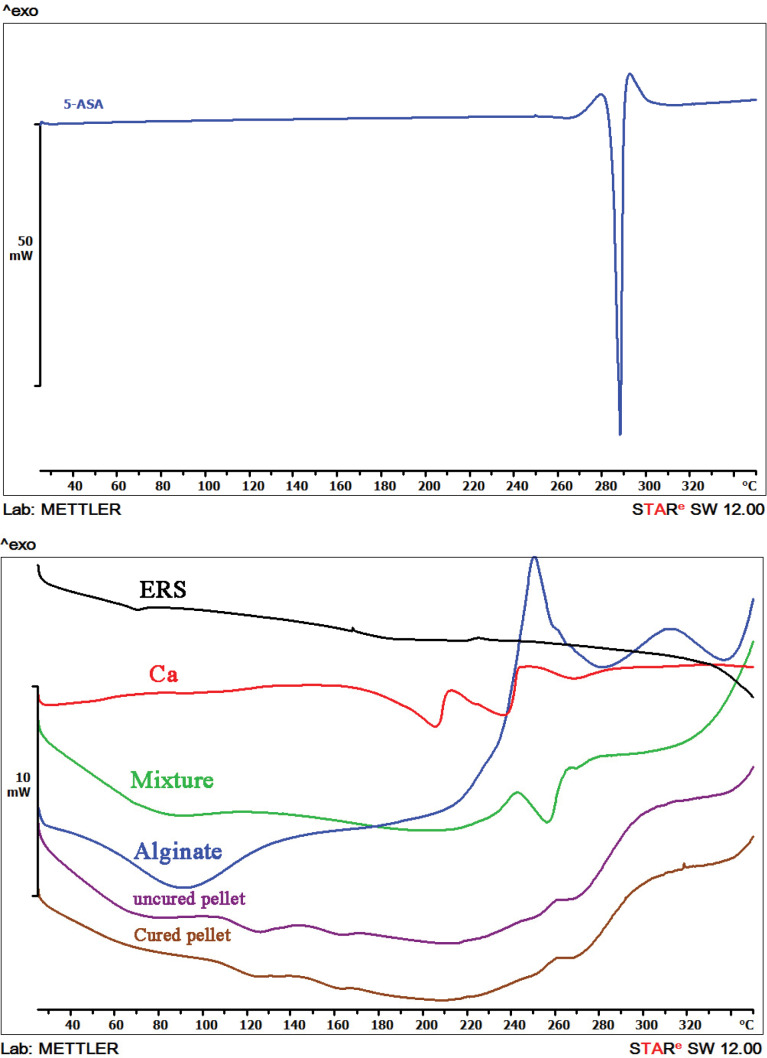
DSC curves of 5-ASA (a) and Eudragit^®^ RS, calcium, sodium alginate, physical mixture, uncured and cured pellets (b).

Due to its crystalline nature, the pure 5-ASA (Figure 3a) showed an endothermic peak at 284.5°C which correlates with the drug melting point. Since the extrusion was conducted at a lower temperature, 5-ASA was dispersed as crystalline particles in the extruded granules.^36^

As can be seen from Figure 3b, an endothermic peak due to the glass transition temperature of Eudragit^®^ RS located at 70.1°C. Endothermic peaks of Ca^2+^ were observed at 205.42°C and 237.28°C that may be corresponded to the dehydration process.^37^ The thermogram of sodium alginate displayed a broad endothermic peak around 80-100°C. An almost flat profile is indicative of the amorphous state of sodium alginate. A sharp exothermic peak at 250.8°C ascribed to a decomposition process.^38^ Physical mixture of 5-ASA, Eudragit^®^ RS and sodium alginate showed a broad endothermic peak around 80.24°C which is may be relevant to the interaction of the glass transition peak of Eudragit^®^ RS and the water loss of moisture content. Replacing the sharp peak of 5-ASA by a broad endothermic peak indicating a reduced melting endotherm at 250-280°C. 5-ASA endothermic peak exhibited a shift to a lower temperature (256.97°C) and a less intensity that could be due to the effect of the alginate. The presence of endothermic peaks in the physical mixture confirmed that 5-ASA crystals still exist in the physical mixture.^39^ There was no difference between the spectrum of the uncured pellet and cured pellet, and both were shown board endometric peaks at about 66°C and 123°C that may be related to the loss of water and restructuring drug from crystalline to amorphous form during the palletization process, respectively.

### FTIR characterization 

**Figure 4:**
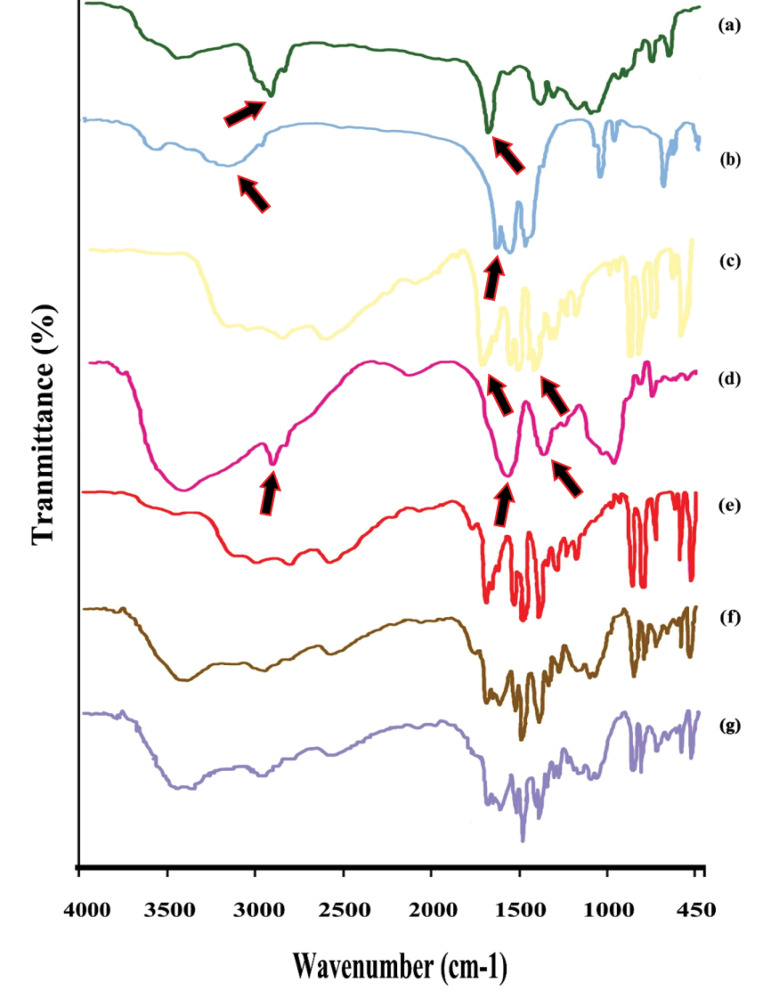
FTIR Spectra of Eudragit^®^ RS (a), Ca^2+^ (b), 5-ASA (c), sodium alginate (d), physical mixture of drug, Eudragit^®^ RS and alginate (e), uncured (f) and cured (g) pellets.

The spectra of Eudragit^®^ RS (Figure 4a) showed a peak at 1738.3 cm^−1^ was attributed to the tertiary amine groups and a peak around 2926 cm^−1^ related to carboxylic acid groups. The spectrum of calcium acetate (Figure 4b) showed peaks in 1611.4 cm^−1^ and 3177.2 cm^−1^. The characteristic peaks of 5-ASA spectra (Figure 4c) related to 1352.8 cm^−1^ peak region (C–N stretch), 1649 cm^−1^ (C=O stretch), 2500-3000 cm^−1^ (stretching vibrations of the hydrogen bonds).^40^ The sodium alginate spectrum (Figure 4d) displayed important absorption bands regarding their functional groups. Stretching vibrations of O–H bonds of alginate appeared in the range of 3000–3500 cm^−1^. Stretching vibrations of aliphatic C–H were observed at 2928 cm^−1^. Observed bands in 1620.8 and 1418.6 cm^−1^ were attributed to asymmetric and symmetric stretching vibrations of carboxylate salt ion, respectively.^41^

No chemical interaction was found between the drug and the polymers (Eudragit^®^ RS and sodium alginate) in the physical mixture used in this study (Figure 4e). Also, the spectrum of cured and uncured pellet formulation (Figure 4f, g) showed no interactions between drugs and excipient FTIR had been used to confirm not any interaction between 5-ASA and excipients.^42,43^

### In vitro drug release studies

**Figure 5:**
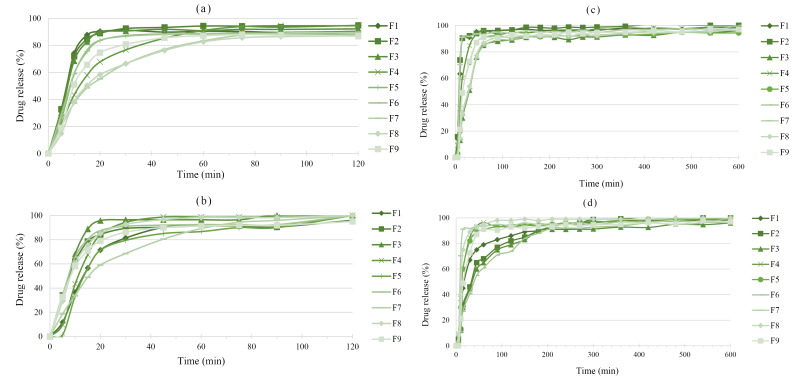
The release of 5-ASA from uncured (a) and cured (b) pellets in 0.1 N HCl, and uncured (c) and cured (d) pellets in phosphate buffer pH 6.8

The swelling dependency of alginate matrices on the pH of the medium is the main characteristic of this polymer that can be used to avoid the gastric release of the drugs via control of their release.^12^ Figure 5 (a and b) shows the release of 5-ASA from uncured and cured pellets in 0.1 N HCl. The curing process only reduced the drug release from F1. Total calcium and alginate amounts in F1 were the highest among all formulations.

After curing, the drug release rate decreases due to the polymer coalescence in the pellet structure and formation of the more coherent matrix around drug particles.^27^ Also, incorporation of alginate into pellets in the presence of calcium ions led to a prolonged release, which is maybe due to the increased number of cross-links. It subsequently reduced swelling after curing.^44^ Similarly, reduced-sodium aceclofenac release from Eudragit RS matrices reported by Suraj et al. after curing at a constant temperature of 60°C.^45^ Moreover, Heat-treating prolonged the release rate of indomethacin from Eudragit RS and RL matrices. The decreased porosity of the matrix may be responsible for this observation.^20^

Drug release was too rapid in phosphate buffer pH 6.8 (Figure 5) due to higher water uptake of alginate matrices at bufferic pH than acidic medium (pH 1.2). The drug release from F5 and F8 formulations was increased after the curing process. Overall, the applied of curing strategy was successful in the F1, F5, and F8 formulations. However, in these formulations, drug release was still too rapid. Hence, in order to reduce the undesired premature drug release, these cured pellets were coated with Eudragit^®^ RS at two levels (5 and 10%). In a study, an undesirable burst dexamethasone release from chitosan-alginate multilayer microcrystals was reported at upper GIT pH values, while coating by the pH-responsive Eudragit S layer provided significant protection against drug dissolution at acidic pH values and sustained drug released at colonic pH.^2^

**Figure 6:**
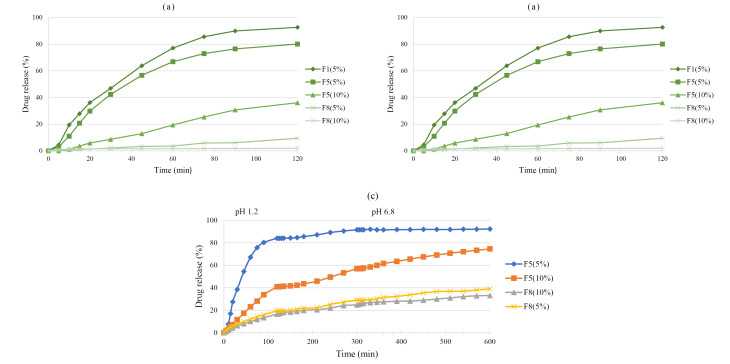
The release of 5-ASA from coated pellets in 0.1 N HCl (a) and phosphate buffer pH 6.8 (b), and Continuous release of 5-ASA from coated pellets (c)

Figure 6 shows 5-ASA release from cured pellets that coated with Eudragit^®^ RS. Figure 6a indicates that all formulations with a higher coating level (10%) had lower drug release in 0.1 N HCl. F8 showed a lower release rate compared to F1 and F5 formulations which may be due to the lower amount of Ca^2+^ and alginate in matrix that reduce the swelling. As can be seen in Figure 6b, both F5 and F8 formulations with lower coating level (5%) showed higher drug release in phosphate buffer pH 6.8. However, the release rate of F5 was higher than F8 due to the higher amount of Ca^2+^ and alginate in its matrix that led to higher swelling of pellets in the buffered medium.^34^ The osmotic pressure gradient between the alginate gel and the environment is an important factor in the swelling. Under acidic stomach conditions, swelling of this system scarcely occurs. A drug may be released by diffusion through the matrix. The drug release under neutral intestine conditions depends on the swelling and erosion process.^46^

Both F5 and F8 formulations with two coating levels (5 and 10%) were selected for studying continuous dissolution in order to provide a realistic *in vitro* simulation of the GIT (Figure 6c). Although both coating levels of F8 formulations had a lower drug release in acidic medium, they failed to release 5-ASA in the colon, and only 36% of the drug was released in this region. This was maybe due to the lower amount of alginate in F8 compared to F5. At pH 6.8, the carboxylic groups of alginate became ionized (COO-), resulting in swelling of the matrix and facilitating the drug release.^47^ F5 formulation with a 5 % coating level showed the highest drug release so that 84 % of 5-ASA was released in 120 min. Expectedly, coating level-up to 10% significantly affected total drug release from Eudragit^®^ RS coated F5 pellets such that the drug release was below 40% in 120 min and showed almost uniform and slower drug release characteristics. Thus F5 (10%) showed more resistance against drug release in acidic stomach medium and also more drug release in ileum basic medium.

## Conclusion

This study presents an approach for the preparation of 5-ASA pellets with the main focus onto the release of the drug into the colon. Pellets possessed proper morphology and mechanical properties selected and coated with Eudragit^®^ RS. The coated pellets showed more efficient drug release into the colon. The slow and consistent drug release from an F5 formulation containing 25% Eudragit^®^ RS, 17.5% alginate, and a coating level of 10% could be exerted to treat the broad range of IBD patients particularly in whom with colonic pH levels of lower than pH 7.0.

## Ethical Issues

This article does not contain any studies with human or animal subjects performed by any of the authors.

## Conflict of Interest

All authors declare that they have no conflict of interest.

## Acknowledgments

The authors would like to appreciate Vice Chancellor for Research and Technology of Mashhad University of Medical Sciences, Mashhad, Iran for the financial support of this study (project code: N-950621).
